# From Quasi-Planar B_56_ to Penta-Ring Tubular Ca©B_56_: Prediction of Metal-Stabilized Ca©B_56_ as the Embryo of Metal-Doped Boron α-Nanotubes

**DOI:** 10.1038/srep37893

**Published:** 2016-11-25

**Authors:** Wen-Juan Tian, Qiang Chen, Xin-Xin Tian, Yue-Wen Mu, Hai-Gang Lu, Si-Dian Li

**Affiliations:** 1 Nanocluster Laboratory, Institute of Molecular Science, Shanxi University, Taiyuan 030006, China; 2 Institute of Materials Science and Department of Chemistry, Xinzhou Teachers’ University, Xinzhou 034000, China

## Abstract

Motifs of planar metalloborophenes, cage-like metalloborospherenes, and metal-centered double-ring tubular boron species have been reported. Based on extensive first-principles theory calculations, we present herein the possibility of doping the quasi-planar *C*_2*v*_ B_56_ (A-1) with an alkaline-earth metal to produce the penta-ring tubular Ca©B_56_ (B-1) which is the most stable isomer of the system obtained and can be viewed as the embryo of metal-doped (4,0) boron α-nanotube Ca©BNT_(4,0)_ (C-1). Ca©BNT_(4,0)_ (C-1) can be constructed by rolling up the most stable boron α-sheet and is predicted to be metallic in nature. Detailed bonding analyses show that the highly stable planar *C*_2*v*_ B_56_ (A-1) is the boron analog of circumbiphenyl (C_38_H_16_) in π-bonding, while the 3D aromatic *C*_4*v*_ Ca©B_56_ (B-1) possesses a perfect delocalized π system over the σ-skeleton on the tube surface. The IR and Raman spectra of *C*_4*v*_ Ca©B_56_ (B-1) and photoelectron spectrum of its monoanion *C*_4*v*_ Ca©B_56_^−^ are computationally simulated to facilitate their spectroscopic characterizations.

It is well known that boron has a strong propensity to form multicenter-two-electron bonds (mc-2e bonds) to compensate for its electron deficiency in both polyhedral molecules and bulk allotropes. Multicenter bonding also appears to dominate the planar or quasi-planar structures of a wide range of gas-phase boron clusters B_*n*_^−/0^ (*n* = 3–25, 27, 30, 35, 36) characterized in a series of combined experimental and theoretical investigations in the past decade^1^,^2^,^3^,^4^,^5^,^6^,^7^,^8^,^9^,^10^,^11^,^12^,^13^. Both the quasi-planar *C*_6*v*_ B_36_^−/0^ 7,8 with a perfect hexagonal hole at the center and the perfect planar *C*_2*v*_ Co©B_18_^−^ with a hepta-coordinate Co were confirmed experimentally to be motifs of the atomically thin planar borophenes and metalloborophenes, respectively. In these flat species, periphery boron atoms are bonded with localized 2c–2e σ bonds along the boundary, while the inner and the periphery atoms are sewed together in blocks with delocalized mc-2e σ or π bonds. Multicenter bonding interactions go extremes from two-dimensional (2D) sheets to three-dimensional (3D) cages in the recently observed borospherenes (all-boron fullerenes) *D*_2*d*_ B_40_^−/0^ 14, *C*_3_/*C*_2_ B_39_^−^ 15, and *C*_2_ B_28_^−/0^ 16 in which all the valence electrons are distributed in delocalized mc-2e σ or π bonds (m ≥ 3). Both endohedral M@B_40_ (M = Ca, Sr) and exohedral M&B_40_ (M=Be, Mg) metalloborospherenes were predicted to be stable species^17^. Two cage-like cations *C*_1_ B_41_^+^ and *C*_2_ B_42_^2+^ predicted at density functional theory (DFT) have also been presented to the B_n_^q^ borospherene family (q = n-40) which are all composed of twelve interwoven boron double chains (BDCs) with six hexagonal or heptagonal faces^18^. Encapsulations of alkaline earth or transition metals have proven to be an effective approach to stabilize borospherene anions like *T*_*h*_ B_36_^4−^ in Li_2_&[Ca@B_36_]^19^, *C*_*s*_ B_37_^3−^ in Ca@B_37_^−^ 20, *C*_*s*_ B_38_^2−^ in Ca@B_38_ 21 and M@B_38_ (M = Sc, Y, Ti)^21^,^22^, and *C*_3_/*C*_2_ B_39_^−^ in Ca@B_39_^+^ 23. The discovery of the first double-ring (DR) tubular B_20_ and the experimental confirmation of the double-ring (DR) tubular B_n_^+^ monocations (n = 16–25)^24^, on the other hand, unveils another important domain in the structural evolution of boron clusters. The recent experimental observation of the metal-centered DR tubular *D*_8*d*_ Co©B_16_^−^ with the coordination of 16 further indicate that doping boron clusters with a transition metal makes an earlier structural transition from 2D planar to 3D tubular in boron clusters^25^. Triple-ring (TR) tubular B_3n_ (n = 8–32) have also been predicted to be competitive isomers at DFT^26^. Very recently, a penta-ring (PR) tubular α-B_84_ with six evenly distributed hexagonal holes in the middle was predicted to be the second lowest-lying isomer of the system at DFT^27^. However, to the best of our knowledge, there have been no PR tubular boron clusters or their metal complexes as the lowest-lying isomers of the systems reported to date. Detailed investigations on the geometrical and electronic structures of multi-ring tubular clusters and their metal-centered complexes may provide key information to understand the geometrical structures and growth mechanisms of the experimentally observed single-walled and multi-walled boron nanotubes (BNTs)^28^,^29^ and the atomically thin borophenes with or without vacancies deposited on Ag(111) substrates^30^,^31^.

Based upon extensive first-principles theory calculations, we present herein the possibility of doping the previously predicted quasi-planar B_56_ (**A-1**)^27^ with an alkaline earth metal to produce the charge-transfer PR tubular complex Ca©B_56_ (**B-1**) which is the most stable isomer of the system obtained and can be viewed as the embryo of metal-doped boron α-nanotube Ca©BNT_(4,0)_ (**C-1**) in a bottom-up approach. Ca©BNT_(4,0)_ (**C-1**) can be constructed by rolling up the most stable boron α-sheet^32^,^33^ and is predicted to be metallic in nature. Detailed molecular orbital analyses show that the quasi-planar *C*_2v_ B_56_ (**A-1**) is analogous to circumbiphenyl (C_38_H_16_) in π bonding, while the PR tubular *C*_4*v*_ Ca^2+^©B_56_^2−^ (**B-1**) possesses a perfect delocalized π system over a σ-skeleton on the tube surface to render tubular aromaticity to the system. The IR, Raman, and photoelectron spectra of the concerned species are simulated to facilitate their experimental characterizations.

## Results and Discussions

**Figure 1:**
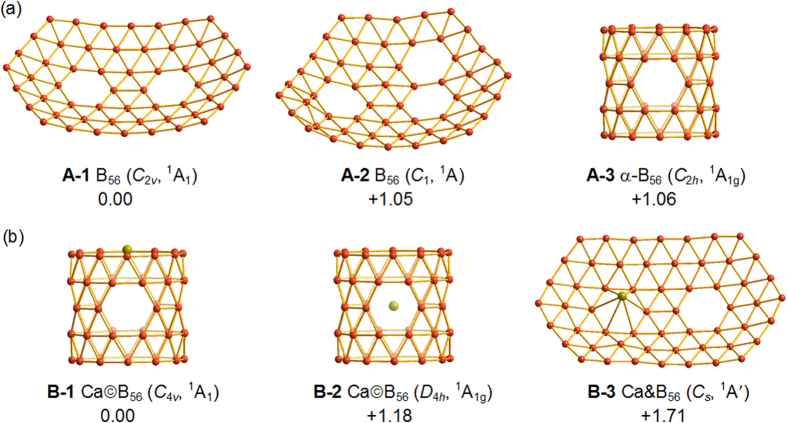
Optimized structures of the three lowest-lying isomers of B_56_ (**a**) and CaB_56_ (**b**), with their relative energies indicated in eV at PBE0/6-311+G(d) level.

We start from the bared B_56_ which may serve as effective ligands to coordinate metal dopants. As shown in Fig. 1(a) and Fig. S1(a), the previously predicted quasi-planar *C*_2*v*_ B_56_ (**A-1**) with two equivalent hexagonal holes^27^ appears to be 1.05 eV and 1.06 eV more stable than the quasi-planar *C*_1_ B_56_ (**A-2**) with three hexagonal holes around the center and the PR tubular *C*_2*h*_ α-B_56_ (**A-3**) with four equivalent hexagonal holes in the middle at PBE0 level, respectively. Other low-lying isomers lie at least 1.19 eV above B_56_ (**A-1**), with the much concerned cage-like borospherene *C*_2_ B_56_ (**A**-**7**) composed of eighteen interwoven BDCs^14^,^15^,^19^,^20^,^21^,^23^ and the irregular cage-like *C*_1_ B_56_ (**A-9**) obtained using first-principles simulated annealing^34^ being 1.78 eV and 2.64 eV less stable, respectively. With two extra electrons, a quasi-planar *C*_2*v*_ B_56_^2−^ similar to B_56_ (**A-1**) appears to be 1.07 eV more stable than the PR tubular *D*_4*h*_ α-B_56_^2−^ similar to α-B_56_ (**A-3**) at PBE0.

With one doping Ca atom added in to compensate for the electron deficiency of the system, surprisingly, a dramatic structural transition occurs from the 2D quasi-planar *C*_2*v*_ B_56_ (**A-1**) to 3D PR tubular *C*_4*v*_ Ca©B_56_ (**B-1**) which has a Ca cap at one end of the tube along the four-fold molecular axis, as demonstrated in Fig. 1(b) and Fig. S1(b). Ca©B_56_ (**B-1**) contains an almost perfect PR tubular *C*_4*v*_ α-B_56_ ligand similar to α-B_56_ (**A-3**) which can be constructed by rolling up the most stable boron α-sheet^32^,^33^. From another perspective of view, Ca©B_56_ (**B-1**) can be viewed as a PR tube composed of interwoven BDCs with four hexagonal holes evenly distributed in the middle, in a structural pattern similar to that of the B_n_^q^ borospherene family (q = n-40, n = 36–42)^14^,^15^,^19^,^20^,^21^,^23^. *C*_4*v*_ Ca©B_56_ (**B-1**) turns out to lie 1.18 eV and 1.71 eV lower than the Ca-centered PR tubular *D*_4*h*_ Ca©B_56_ (**B-2**) based on α-B_56_ (**A-3**) and the face-capped quasi-planar *C*_*s*_ CaB_56_ (**B-3**) based on *C*_2*v*_ B_56_ (**A-1**), respectively. Other low-lying planar, tubular, or cage-like isomers appear to be at least 1.82 eV less stable than Ca©B_56_ (**B-1**), with the endohedral metalloborospherenes *S*_4_ Ca@B_56_ (**B-6**) based on B_56_ (**A**-**7**) and *C*_1_ Ca@B_56_ (**B-13**) based on B_56_ (**A**-**9**) lying 1.87 eV and 2.29 eV higher, respectively. These large relative energies provide strong evidence that Ca©B_56_ (**B-1**) is the first PR tubular species as the most stable isomer of the system obtained to date. We notice that *C*_4*v*_ Ca©B_56_ (**B-1**) possesses two small degenerate imaginary vibrational frequencies at 38.5i cm^−1^ at PBE0 (*e* modes) which lead to the slightly distorted PR tubular *C*_*s*_ Ca©B_56_ (**B-4**) when fully relaxed. However, the energy difference between *C*_4*v*_ Ca©B_56_ (**B-1**) and *C*_*s*_ Ca©B_56_ (**B-4**) turns out to be only 0.01 eV with zero-point corrections included, strongly suggesting that they correspond to the same tubular isomer possible to exist in experiments, given the accuracy of the DFT-PBE0 method employed. Ca©B_56_ (**B-1**) is thus the vibrationally averaged structure of PR tubular CaB_56_, with the Ca atom slightly off-centered circling around the molecular axis of the PR tube on the top.

Such a structural transition from 2D planar to 3D PR tubular also occurs to SrB_56_ at PBE0 (see Fig. S2, the Stuttgart relativistic small-core pseudopotential and valence basis set was used for Sr^35^,^36^). Natural bonding orbital (NBO) analyses indicate that the PR tubular *C*_4*v*_ Ca©B_56_ (**B-1**) and *C*_4*v*_ Sr©B_56_ possess the natural atomic charges of q_Ca_ = +1.83|e| and q_Sr_ = +1.85|e| and the corresponding electronic configurations of Ca[Ar]4s^0.07^3d^0.09^ and Sr[Kr]5s^0.06^4d^0.07^, respectively. They are therefore typical charge-transfer complexes *C*_4*v*_ Ca^2+^©B_56_^2−^ (**B-1**) and *C*_4*v*_ Sr^2+^©B_56_^2−^ in nature in which the alkaline earth metal donates two *ns*^*2*^ valence electrons to the PR tubular α-B_56_ acceptor. Such complexes are mainly stabilized by effective electrostatic interactions between the M^2+^ dication (M = Ca, Sr) and the PR tubular α-B_56_^2−^ ligand. Weak *π→d* back donations from the α-B_56_^2−^ ligand to the M^2+^ metal center may also contribute to stabilize the complexes, as suggested by the electronic configurations mentioned above, similar to the situation in M@B_40_ (M = Ca, Sr)^17^.

Extensive molecular dynamics simulations using the CP2K program^37^ indicate that Ca©B_56_ (**B-1**) is dynamically stable at both 600 K and 800 K, with the root-mean-square-deviations of RMSD = 0.18 Å and 0.18 Å and the maximum bond length deviations of MAXD = 1.10 Å and 1.13 Å, respectively (Fig. S3). We notice that, although the tubular α-B_56_ ligand experiences more or less structural deformations during MD simulations, the Ca cap in Ca©B_56_ (**B-1**) keeps almost still at the center of the top B_12_ ring in the simulation processes. MD simulations indicate that Ca©B_56_ (**B-1**) remains dynamically stable even at 1000 K, with RMSD = 0.19 Å and MAXD = 1.14 Å, respectively, further indicating its high dynamical stability.

**Figure 2:**
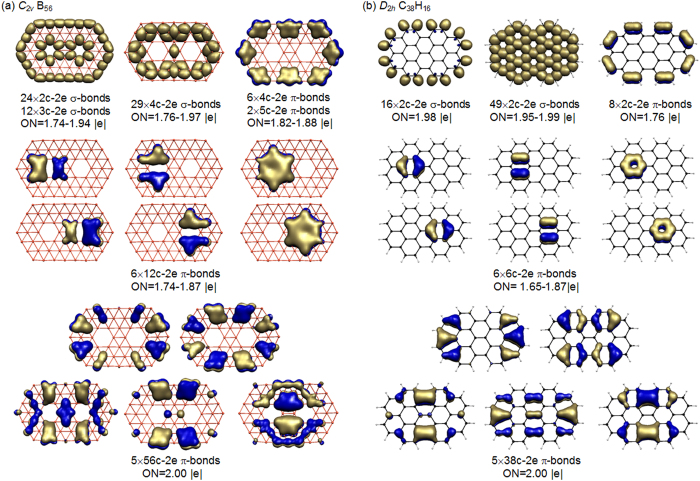
AdNDP bonding patterns of *C*_2*v*_ B_56_ (**1**) (**a**) compared with that of the *D*_2*h*_ circumbiphenyl C_38_H_16_ (**b**), with the occupation numbers (ON) indicated.

The high stability of planar *C*_2*v*_ B_56_ (**A-1**) originates from its electronic structure and bonding pattern. As analyzed in Fig. 2 using the adaptive natural density partitioning (AdNDP) approach^38^, *C*_2*v*_ B_56_ (**A-1**) possesses 24 2c-2e localized σ bonds between neighboring periphery B atoms along the boundary, 12 3c-2e delocalized σ bonds on 12 B_3_ triangles around the two hexagonal holes, and 29 4c-2e delocalized σ bonds on 29 symmetrically distributed B_4_ rhombuses. The remaining 19 π bonds can be classified into four sets, with one set with 6 4c-2e π bonds and 2 5c-2e π bonds along the periphery, two sets with 3 12c-2e π bonds over the hexagonal hole on the right and 3 12c-2e π bonds over the hexagonal hole on the left, and one set with 5 56c-2e π bonds over the whole molecular surface, in the overall symmetry of *C*_2*v*_. *C*_2v_ B_56_ (**A-1**) thus contains two local π-aromatic systems around the two hexagonal holes and one global π-aromatic system over the whole molecular plane which have a one-to-one correspondence with the aromatic π-systems of circumbiphenyl (C_38_H_16_) (see Fig. 2). It is therefore the boron analog of *D*_2*h*_ C_38_H_16_, the biggest boron analog of aromatic polycyclic hydrocarbon reported to date^1^,^2^,^3^,^4^,^5^,^6^,^7^,^8^,^9^,^10^,^11^,^12^,^13^,^14^,^15^,^16^.

**Figure 3:**
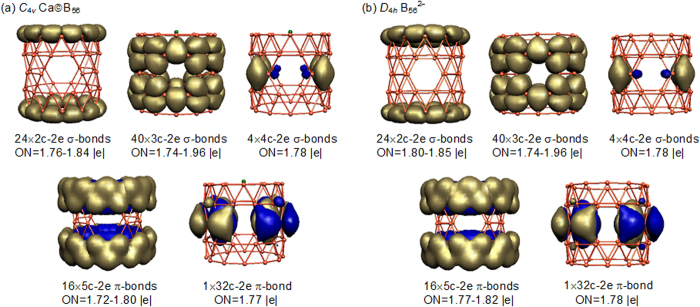
AdNDP bonding patterns of *C*_4*v*_ CaB_56_ (**a**) compared with that of *D*_4*h*_ B_56_^2−^ (**b**) with the occupation numbers indicated.

The bonding pattern of PR tubular Ca©B_56_ (**B-1**) is more unique and intriguing. As shown in Fig. 3, it has 24 2c-2e localized σ bonds between neighboring periphery boron atoms on the top and bottom of the tube with the occupation numbers of ON = 1.76–1.84 |e|, 40 3c-2e delocalized σ bonds on the 40 inner B_3_ triangles with ON = 1.74–1.96 |e|, and 4 4c-2e delocalized σ bonds in the middle on the four uncovered B_4_ rhombuses between neighboring hexagonal holes with ON = 1.78 |e|. The σ-skeleton on the tube surface thus contains 68 σ bonds in total. The remaining 34 valence electrons form a perfectly delocalized π system over the delocalized σ-skeleton, with 16 5c–2e π bonds evenly distributed over two B_24_ DR tubular subunits at the two ends of the tube with ON = 1.72–1.80 |e| and 1 32c-2e π bond over the four evenly distributed hexagonal holes between the two subunits with ON = 1.77 |e|, in the overall symmetry of C_4v_. As shown in Fig. 3, the bared PR tubular *D*_4*h*_ α-B_56_^2−^ dianion possesses the same σ and π bonding patterns as *C*_4*v*_ Ca©B_56_ (**B-1**), further supporting the charge transfer of two 4 s electrons from Ca atom to the tubular α-B_56_ ligand. The AdNDP bonding patterns of *C*_4*v*_ Ca©B_56_ (**B-1**) and *D*_4*h*_α-B_56_^2−^ with ON ≥ 1.72 |e| match the chemical intuitions for tubular boron clusters which can be constructed by rolling up the α-sheet^32^,^33^. Such bonding patterns render 3D tubular aromaticities to the systems, as evidenced by the huge negative NICS values of −44.6 ppm and −46.0 ppm at the tube centers calculated for *C*_4*v*_ Ca©B_56_ (**B-1**) and *D*_4*h*_ α-B_56_^2−^, respectively.

**Figure 4:**
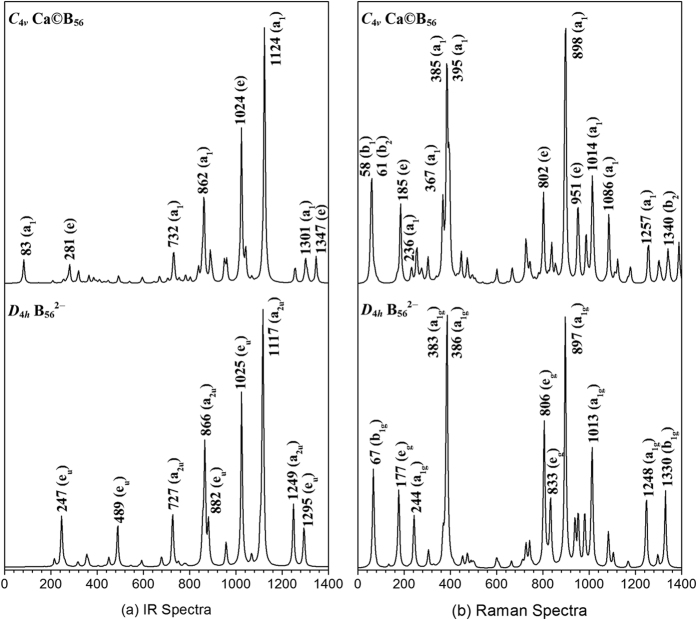
Simulated IR (**a**) and Raman (**b**) spectra of *C*_4*v*_ Ca©B_56_ (**B-1**) compared with that of the bared PR tubular *D*_4*h*_ B_56_^2−^.

The IR and Raman spectra of *C*_4*v*_ Ca©B_56_ (**B-1**) are computationally simulated and compared with that of the perfect PR tubular *D*_4*h*_ α-B_56_^2−^ in Fig. 4 to facilitate its future spectroscopic characterization. As expected, the major IR peaks at 1249 cm^−1^ (a_2u_ mode), 1117 cm^−1^ (a_2u_), 1025 cm^−1^ (e_u_), and 866 cm^−1^ (a_2u_) in *D*_4*h*_ α-B_56_^2−^ are all basically maintained in *C*_4*v*_ Ca©B_56_ (**B-1**). The major Raman features at 1248 cm^−1^ (a_1g_), 1013 cm^−1^ (a_1g_), 897 cm^−1^ (a_1g_), 806 cm^−1^ (e_g_), and 386 cm^−1^ (a_1g_) in *D*_4*h*_ α-B_56_^2−^ also appear in *C*_4*v*_ Ca©B_56_ (**B-1**). The breathing modes at 236 cm^−1^ (a_1_) in *C*_4*v*_ Ca©B_56_ (**B-1**) and 244 cm^−1^ (a_1g_) in *D*_4*h*_ α-B_56_^2−^ belong to typical “radial breathing modes” (RBMs) of the tubular species which may help characterize metal-centered boron α-nanotubes in future experiments. A strong RBM band observed at 210 cm^−1^ was used to identify the hollow structures of the single-walled boron nanotubes^28^.

**Figure 5:**
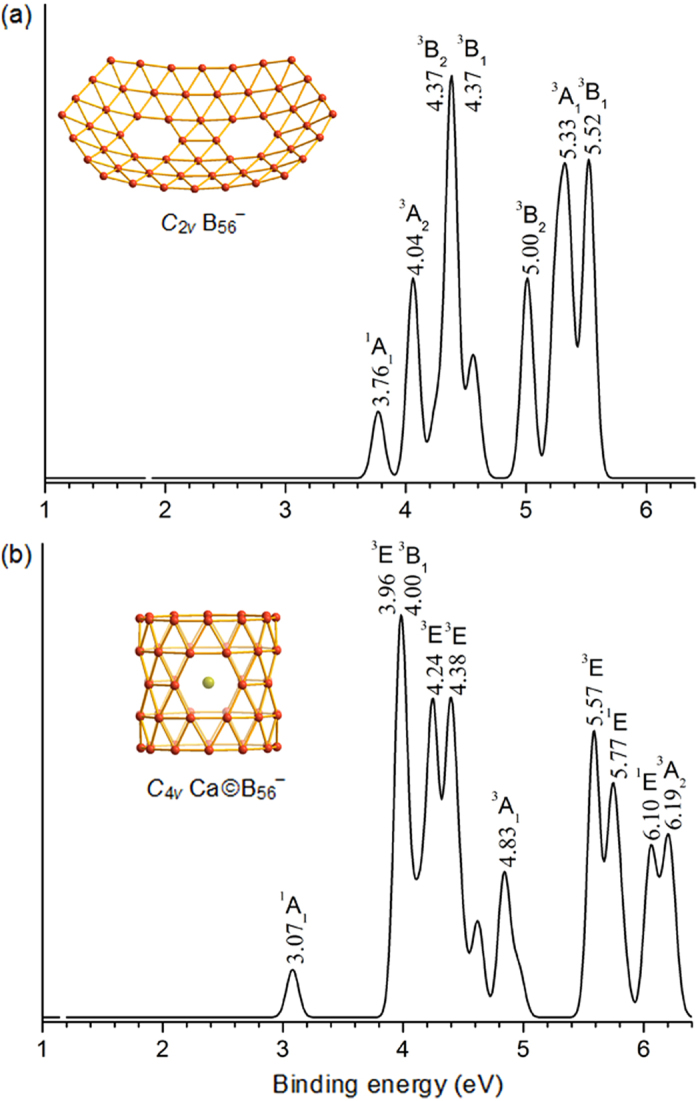
Simulated PES spectra of *C*_2*v*_ B_56_^−^ (**a**) and *C*_4*v*_ Ca©B_56_^−^ (**b**) at PBE0.

**Figure 6:**
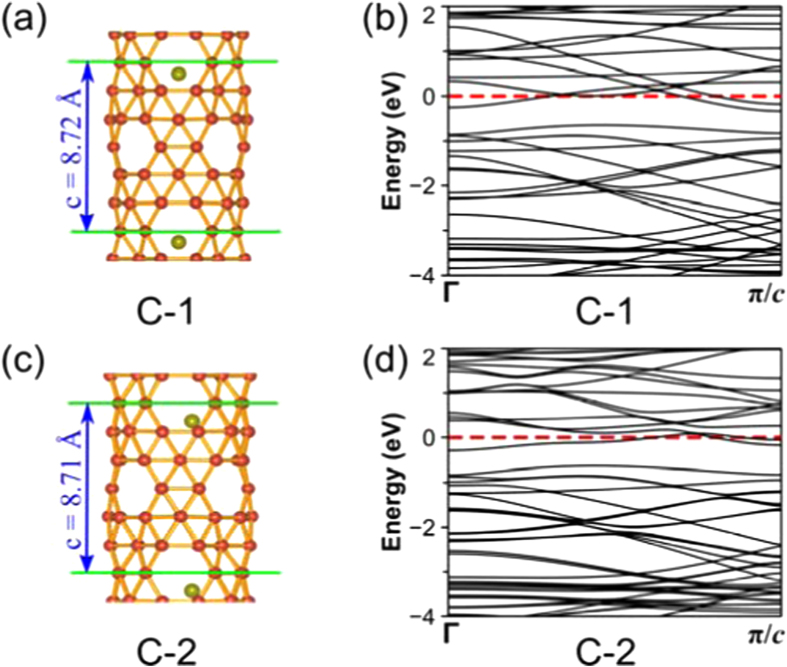
Optimized geometries (**a** and **c**) and band structures (**b** and **d**) of Ca©BNT_(4,0)_ with *P*_4_ (*C*_4_) symmetry and Ca©BNT_(4,0)_ with *P*_1_ (*C*_1_) symmetry, with the lattice parameters *c* in *z* direction indicated.

Combination of the PES spectra and first-principles theory calculations has proven to be the most powerful approach to characterize novel boron clusters over the past decade^1^,^2^,^3^,^4^,^5^,^6^,^7^,^8^,^9^,^10^,^11^,^12^,^13^,^14^,^15^,^16^,^25^. We calculate the vertical excitation energies and simulate the PES spectra of the corresponding monoanions of *C*_2*v*_ B_56_ (**A-1**) and *C*_4*v*_ Ca©B_56_ (**B-1**) at PBE0 level in Fig. 5. As shown in Fig. 5(a), quasi-planar *C*_2*v*_ B_56_^−^, the monoanion of *C*_2*v*_ B_56_ (**A-1**), possesses a high first vertical detachment energy of VDE = 3.76 eV and a simulated PES spectrum similar to that of the observed quasi-planar *C*_*s*_ B_40_^−^ which has the first VDE of 3.60 eV^14^. PR tubular *C*_4*v*_ Ca©B_56_^−^, the monoanion of *C*_4*v*_ Ca©B_56_ (**B-1**), appears to possess a PES spectrum with a low VDE of 3.07 eV and a large energy gap of 0.89 eV (Fig. 6(b)), in line with the large HOMO-LUMO energy gap of 2.00 eV calculated for tubular Ca©B_56_ (**B-1**) at PBE0.

Finally, using *C*_4*v*_ Ca©B_56_ (**B-1**) as an embryo at the center of the unit cell, we construct the metal-doped (4,0) boron α-nanotube Ca©BNT_(4,0)_ (**C-1**) in a bottom-up approach which has a *P*_4_ symmetry as shown in Fig. 6(a). A similar approach was used to predict the nanotubes of silicon^39^. Ca©BNT_(4,0)_ (**C-1**) turns out to possess the optimized diameter of 6.55 Å and the lattice parameter of c = 8.72 Å in z direction (Fig. 6(a)). Interestingly, as indicated in its calculated band structures in Fig. 6(b), with several bands crossing the Fermi level, Ca©BNT_(4,0)_ (**C-1**) is predicted to be typically metallic in nature, in strong contrast to the bared α-boron nanotube BNT_(4,0)_ which is a semiconductor with the band gap of 0.69 eV at PBE level (Fig. S4) (0.75 eV at GGA^33^), indicating that the transport properties of boron nanotubes can be dramatically changed by metal-doping. It is noticed that the slightly off-centered Ca-doped boron α-nanotube Ca©BNT_(4,0)_ (**C-2**) with *P*_1_ symmetry (Fig. 6(c)) possesses a total energy slightly lower than Ca©BNT_(4,0)_ (**C-1**) (by 0.22 eV per unit cell). However, the two metallic Ca-doped boron α-nanotube with slightly different geometries (Fig. 6(a and c)) possess very similar band structures (Fig. 6(b and d)), indicating that minor changes in positions of the metal donors cause no obvious changes in both the geometries and conductivities of metal-doped boron α-nanotubes.

In summary, we have performed in this work an extensive first-principles theory investigation on the possibility of doping the quasi-planar *C*_2*v*_ B_56_ (**A-1**) with an alkaline-earth metal to produce the 3D aromatic PR tubular Ca©B_56_ (**B-1**) which is the most stable isomer obtained and can be viewed as the embryo of the metal-doped boron α-nanotube Ca©BNT_(4,0)_ (**C-1**). The high stability of the 3D aromatic *C*_4*v*_ Ca©B_56_ (**B-1**) originates from its unique bonding pattern which possesses a perfect delocalized π system over a σ-skeleton on the tube surface. Metal dopants encapsulated in cage-like borospherenes to form metalloborospherenes^17^,^19^,^20^,^21^,^22^,^23^, inserted in planar borophenes to form metalloborophenes^9^, or wrapped up in boron nanotubes to form metal-doped boron nanotubes may effectively enhance the chemical stabilities and tune the transport properties of the boron nanostructures. Metal-stabilized boron nanostructures are expected to be complementary with the corresponding carbon nanomaterials in applications and warrant further theoretical and experimental investigations.

### Theoretical procedures

Initial structures were constructed for CaB_56_ based on the previously reported lowest-lying planar or cage-like isomers of B_56_^27^,^34^. Cage-like borospherene structures composed of interwoven BDCs were also built for B_56_ according to the structural pattern of borospherenes^14^,^15^,^19^,^20^,^21^,^23^. In particular, a PR tubular α-B_56_ (**A-2**) with four hexagonal holes evenly distributed in the middle was constructed by rolling up the most stable boron α-sheet^32^,^33^. Low-lying isomers thus obtained were then fully optimized with frequencies checked at the hybrid DFT-PBE0^40^ level with the basis set of 6–311+G(d)^41^ implemented in Gaussian 09 suite^42^. Minima Hopping^43^ searches with over 500 stationary points probed produced no isomers with lower energies than Ca©B_56_ (**B-1**) (see Fig.1 and S1(b)). The bonding patterns of the quasi-planar *C*_2*v*_ B_56_ (**A-1**) (Fig. 2) and PR tubular *C*_4*v*_ Ca©B_56_ (**B-1**) (Fig. 3) were analyzed using the AdNDP method which is an extension of the Lewis valence bond theory to include multicenter mc-2e interactions (m ≥ 3)^38^ and has been successfully applied to various systems^8^,^9^,^10^,^11^,^12^,^13^,^14^,^15^,^16^,^17^,^18^,^19^,^20^,^21^,^23^,^25^. The photoelectron spectroscopy (PES) of the quasi-planar *C*_2*v*_ B_56_^−^ and PR tubular *C*_4*v*_ Ca©B_56_^−^ were simulated using the time-dependent DFT (TD-DFT-PBE0) approach^44^. The metal-centered α-boron nanotube Ca©BNT_(4,0)_ (**C-1**) constructed by rolling up the most stable boron α-sheet^32^,^33^ was optimized using Vienna *ab initio* simulation package (VASP)^45^,^46^ at Perdew-Burke-Ernzerhof (PBE)^47^ level with the projector augmented wave (PAW)^48^,^49^ pseudopotential method. The nucleus-independent chemical shift (NICS)^50^ values at the tube centers of Ca©B_56_ (**B-1**) and *D*_4*h*_ B_56_^2−^ were calculated to assess their tubular aromaticities.

## Additional Information

**How to cite this article**: Tian, W.-J. *et al*. From Quasi-Planar B_56_ to Penta-Ring Tubular Ca©B_56_: Prediction of Metal-Stabilized Ca©B_56_ as the Embryo of Metal-Doped Boron α-Nanotubes. *Sci. Rep.* **6**, 37893; doi: 10.1038/srep37893 (2016).

**Publisher’s note:** Springer Nature remains neutral with regard to jurisdictional claims in published maps and institutional affiliations.

## Supplementary Material

Supplementary Information
