# Supplementary material for: From Quasi-Planar B56 to Penta-Ring Tubular Ca©B56: Prediction of Metal-Stabilized Ca©B56 as the Embryo of Metal-Doped Boron α-Nanotubes
Source: Sci Rep. 2016 Nov 25;6:37893. doi: 10.1038/srep37893 (PMC5122883; doi:10.1038/srep37893)
Supplement: Supplementary Information [file srep37893-s1.doc]

**Supporting Information**

**From Quasi-Planar B_56_ to Penta-Ring Tubular Ca©B_56_: Prediction of Metal-Stabilized Ca©B_56_ as the Embryo of Metal-Doped Boron α-Nanotubes**

Wen-Juan Tian,^1^ Qiang Chen,^1,2^ Xin-Xin Tian,^1^ Yue-Wen Mu,^1,*^ Hai-Gang Lu,^1,*^and Si-Dian Li^1,*^

^1^*Nanocluster Laboratory,* *Institute of Molecular Science, Shanxi University, Taiyuan 030006, China*

*^2^ Institute of Materials Science and Department of Chemistry,* *Xinzhou Teachers’ University, Xinzhou 034000, China*

Electronic mail: [ywmu@sxu.edu.cn](mailto:ywmu@sxu.edu.cn); [luhg@sxu.edu.cn](mailto:luhg@sxu.edu.cn); and [lisidian@sxu.edu.cn](mailto:lisidian@sxu.edu.cn).

**Figure S1**. Optimized low-lying isomers of B_56_ (**a**) and CaB_56_ (**b**), with their relative

energies indicated in eV at PBE0/6-311+G* level.


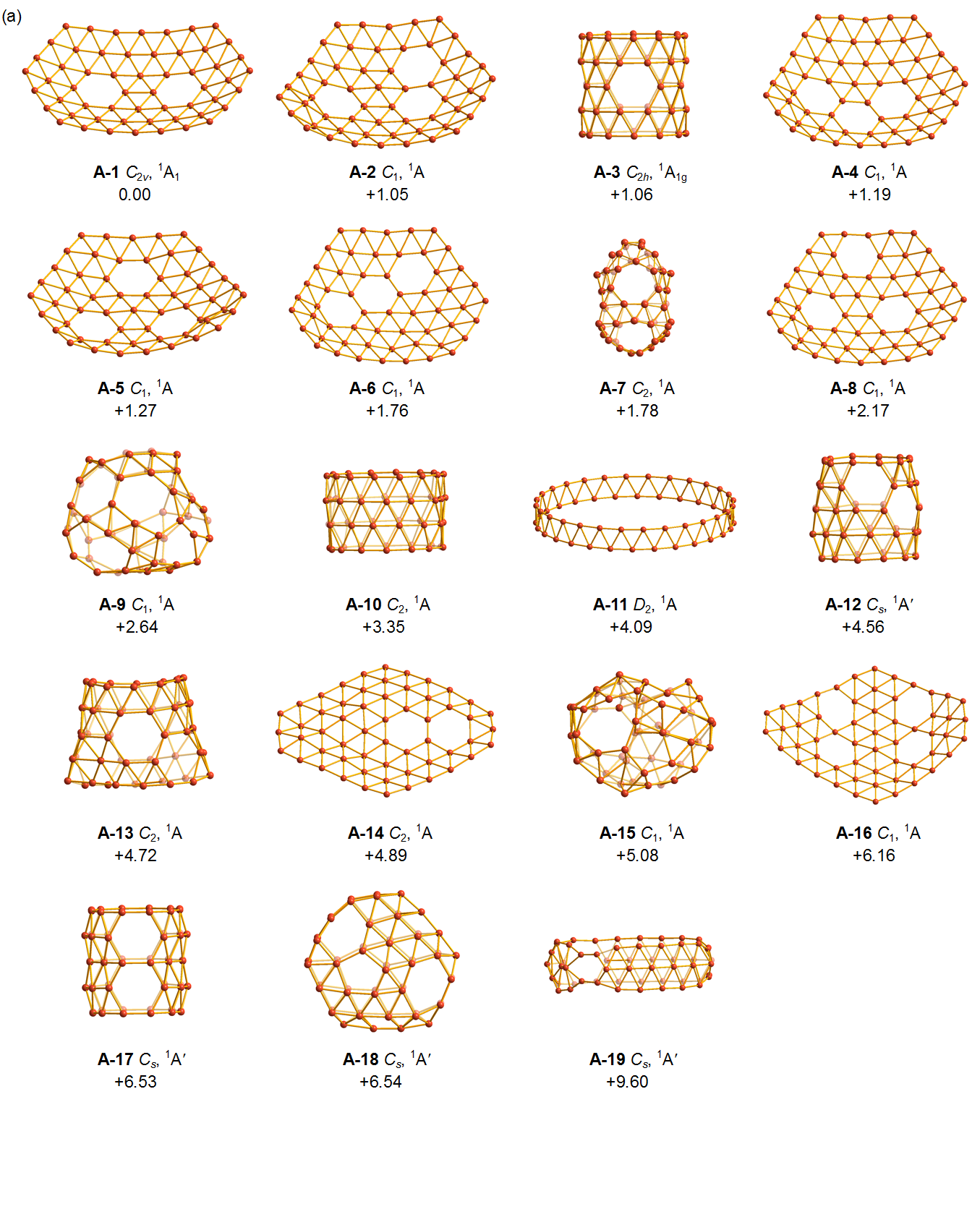


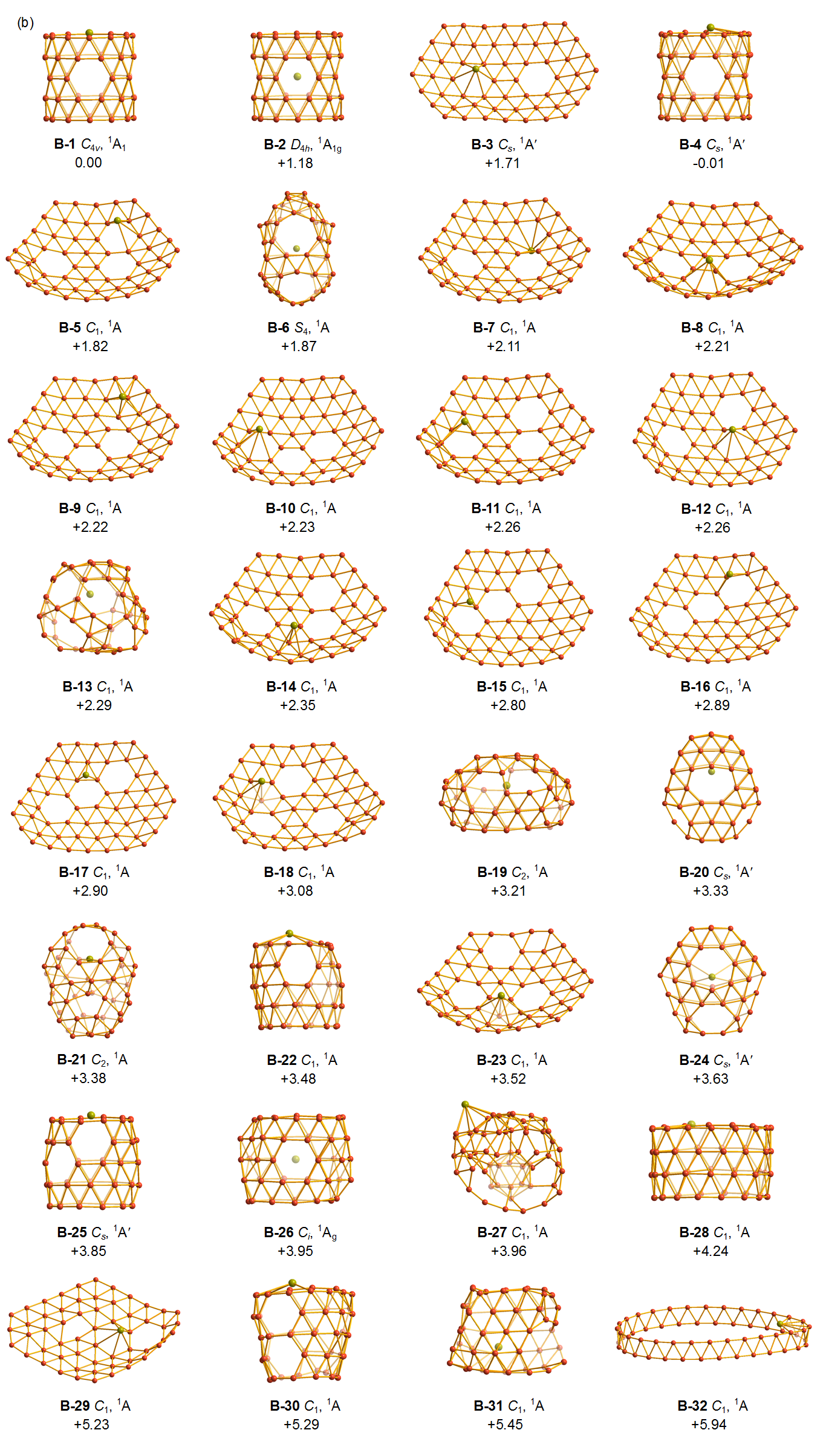


**Figure S2**. Optimized low-lying isomers of SrB_56_, with their relative energies

indicated in eV at PBE0/Sr/stuttgart/B/6-311+G* level.


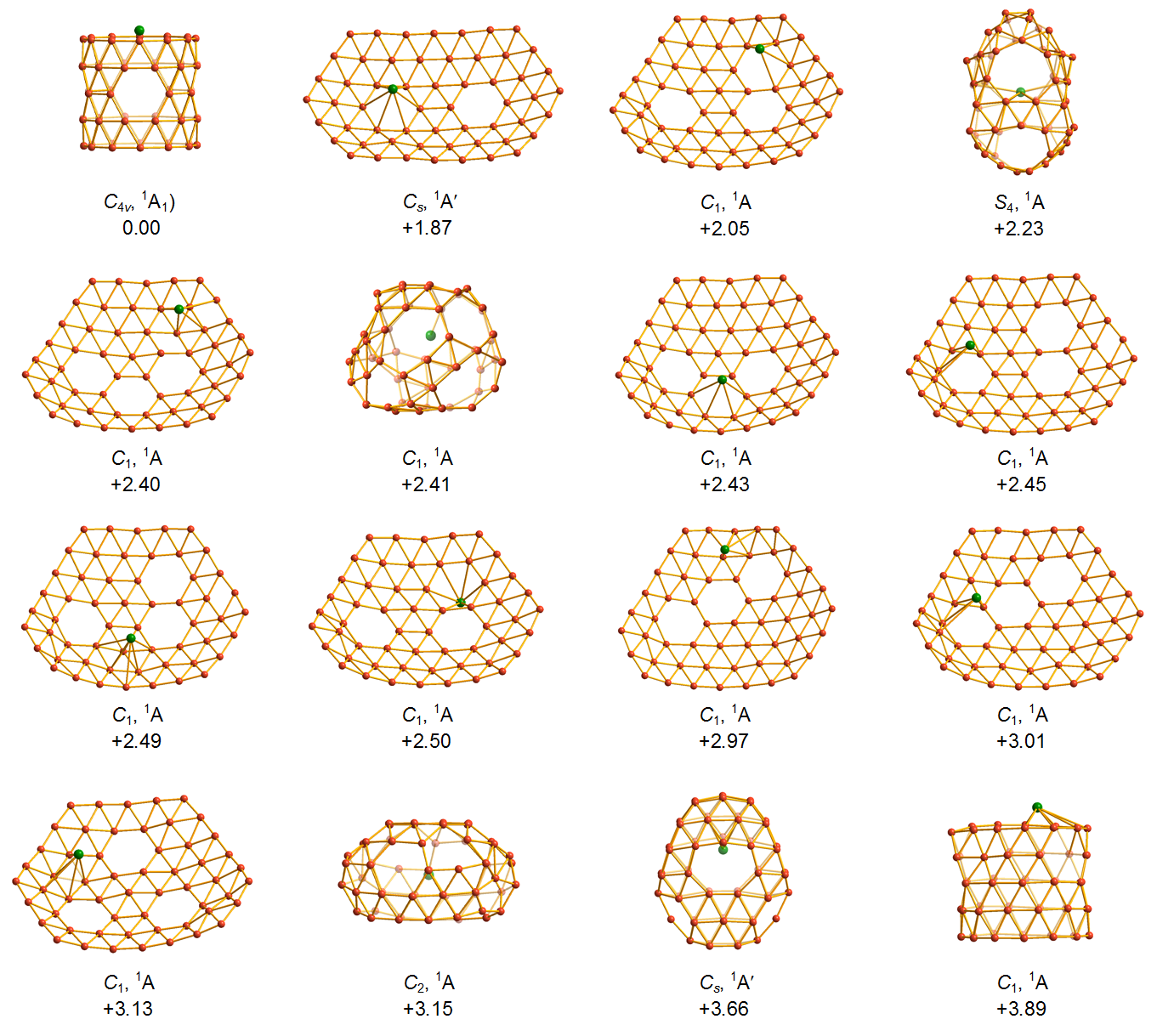


**Figure S3**. Born-Oppenheimer molecular dynamics simulations of *C*_4_*_v_* Ca©B_56_ at (a) 600K, (b) 800k and (c) 1000K for 30 ps using the software suite CP2K (The CP2K developers group, 2000−2011, <http://cp2k.berlios.de/>),

with the root-mean-square-deviation (RMSD) and maximum bond length

deviation (MaxD) values (on average) indicated in Å.

**
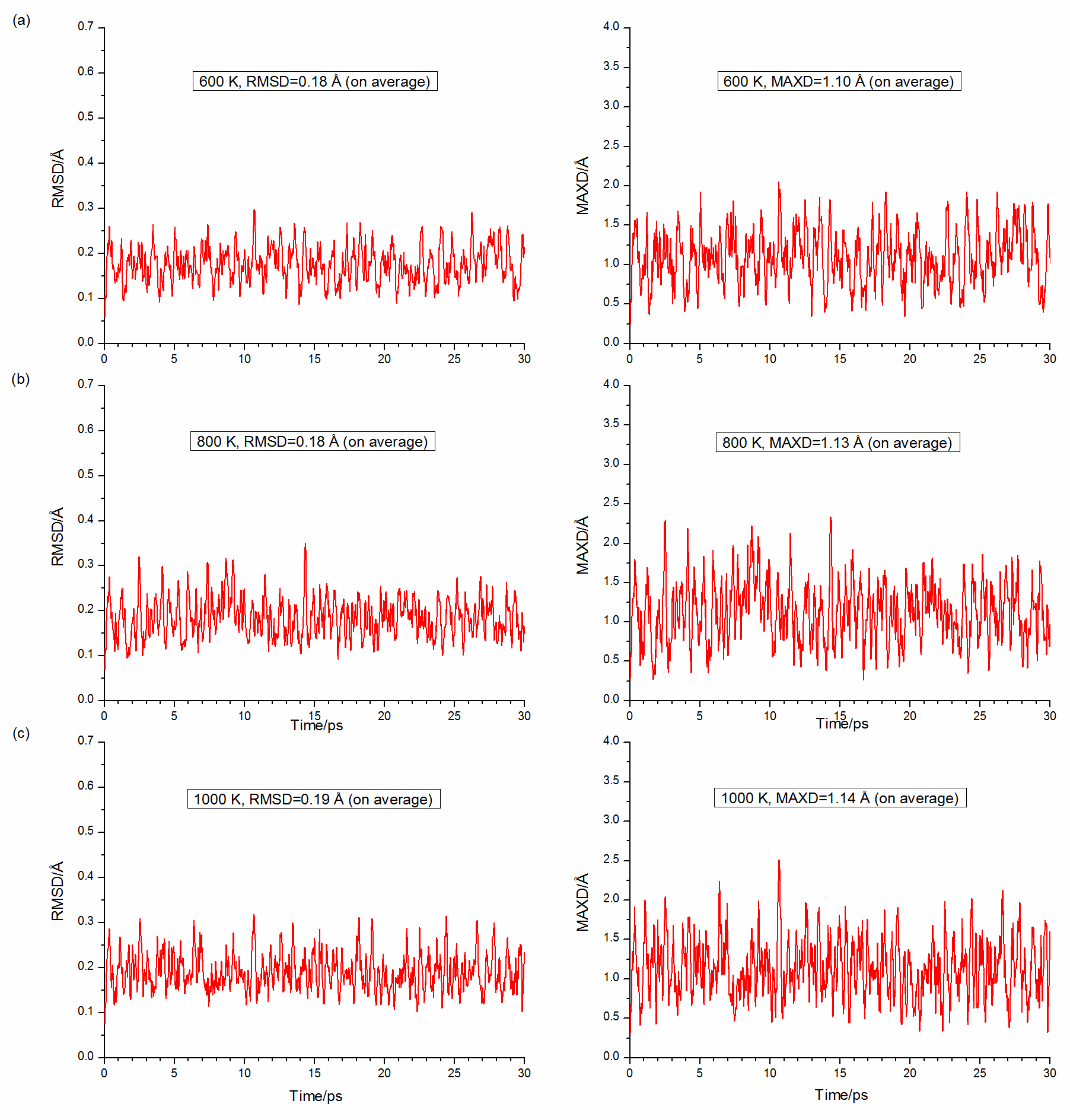
**

**Figure S4**. Geometry (a) and band structure (b) of α-BNT_(4,0)_.

**
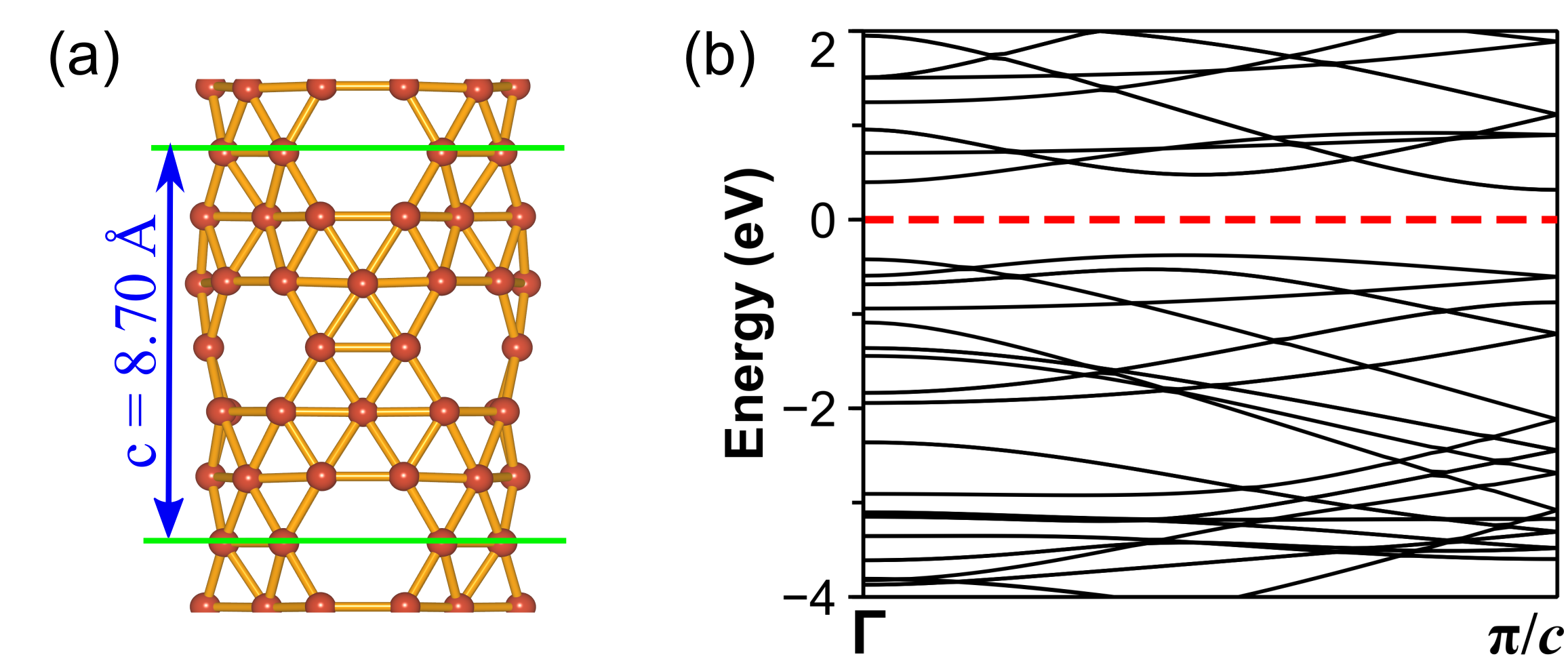
**

**Table S1**. Optimized coordinates ( (x, y, z) in Å) of *C_2v_* B_56_ (**A-1**), *C*_4_*_v_* Ca©B_56_ (**B-1**), *D*_4_*_h_* Ca©B_56_ (**B-2**), *C_s_* Ca©B_56_ (**B-4**), and *D*_4_*_h_* B_56_^2−^ at PBE0/6-311+G* level.

| *C*_2_*_v_* B_56_ (**A-1**) |
| --- |

B 1.43168500 1.67760600 -0.82183700

B 1.44541400 3.32931500 -0.59445400

B 0.00000000 4.14649400 -0.46427200

B -1.44541400 3.32931500 -0.59445400

B -1.43168500 1.67760600 -0.82183700

B 0.00000000 0.88853300 -0.91722200

B -2.91329700 0.83047100 -0.51218000

B -2.85446100 4.10652900 0.01488700

B 2.91329700 0.83047100 -0.51218000

B -2.88366200 2.47593000 -0.33055800

B 2.88366200 2.47593000 -0.33055800

B -1.43224100 4.92370300 -0.05678200

B -4.29634800 3.23770000 0.40086700

B -4.28764900 1.61952500 0.07368700

B -1.43168500 -1.67760600 -0.82183700

B -2.88366200 -2.47593000 -0.33055800

B -4.28764900 -1.61952500 0.07368700

B -4.29634800 -3.23770000 0.40086700

B -2.85446100 -4.10652900 0.01488700

B -1.44541400 -3.32931500 -0.59445400

B -1.43224100 -4.92370300 -0.05678200

B 1.44541400 -3.32931500 -0.59445400

B 0.00000000 -4.14649400 -0.46427200

B 1.43168500 -1.67760600 -0.82183700

B 2.88366200 -2.47593000 -0.33055800

B 2.91329700 -0.83047100 -0.51218000

B -4.33448100 0.00000000 0.00275200

B 0.00000000 -0.88853300 -0.91722200

B 4.28764900 1.61952500 0.07368700

B 4.33448100 0.00000000 0.00275200

B 4.28764900 -1.61952500 0.07368700

B -2.91329700 -0.83047100 -0.51218000

B 0.00000000 5.72459900 0.20308300

B 1.43224100 4.92370300 -0.05678200

B 2.85446100 4.10652900 0.01488700

B 4.29634800 3.23770000 0.40086700

B 0.00000000 -5.72459900 0.20308300

B 1.43224100 -4.92370300 -0.05678200

B 2.85446100 -4.10652900 0.01488700

B 4.29634800 -3.23770000 0.40086700

B -1.45107900 0.00000000 -0.92871600

B 1.45107900 0.00000000 -0.92871600

B -4.14591300 -4.75737100 0.81203200

B -2.86051700 -5.65717300 0.71261500

B -1.41367800 -6.48862100 0.81283700

B 0.00000000 -7.12100600 1.08214300

B 1.41367800 -6.48862100 0.81283700

B 2.86051700 -5.65717300 0.71261500

B 4.14591300 -4.75737100 0.81203200

B 4.14591300 4.75737100 0.81203200

B 2.86051700 5.65717300 0.71261500

B 1.41367800 6.48862100 0.81283700

B 0.00000000 7.12100600 1.08214300

B -1.41367800 6.48862100 0.81283700

B -2.86051700 5.65717300 0.71261500

B -4.14591300 4.75737100 0.81203200

| *C*_4_*_v_* Ca©B_56_ (**B-1**) |
| --- |

B 0.79461000 2.95988700 2.75123800

B -0.79461000 2.95988700 2.75123800

B -2.18904200 2.18904200 2.74143300

B -2.95988700 0.79461000 2.75123800

B -2.95988700 -0.79461000 2.75123800

B -2.18904200 -2.18904200 2.74143300

B -0.79461000 -2.95988700 2.75123800

B 0.79461000 -2.95988700 2.75123800

B 2.18904200 -2.18904200 2.74143300

B 2.95988700 -0.79461000 2.75123800

B 2.95988700 0.79461000 2.75123800

B 2.18904200 2.18904200 2.74143300

B 1.63767000 2.82944500 1.25796500

B 0.00000000 3.19874800 1.26253900

B -1.63767000 2.82944500 1.25796500

B -2.82944500 1.63767000 1.25796500

B -3.19874800 0.00000000 1.26253900

B -2.82944500 -1.63767000 1.25796500

B -1.63767000 -2.82944500 1.25796500

B 0.00000000 -3.19874800 1.26253900

B 1.63767000 -2.82944500 1.25796500

B 2.82944500 -1.63767000 1.25796500

B 3.19874800 0.00000000 1.26253900

B 2.82944500 1.63767000 1.25796500

B 0.84968300 3.15454800 -0.20426100

B -0.84968300 3.15454800 -0.20426100

B -3.15454800 0.84968300 -0.20426100

B -3.15454800 -0.84968300 -0.20426100

B -0.84968300 -3.15454800 -0.20426100

B 0.84968300 -3.15454800 -0.20426100

B 3.15454800 -0.84968300 -0.20426100

B 3.15454800 0.84968300 -0.20426100

B 1.63627100 2.83310500 -1.68714300

B 0.00000000 3.21377900 -1.71368500

B -1.63627100 2.83310500 -1.68714300

B -2.83310500 1.63627100 -1.68714300

B -3.21377900 0.00000000 -1.71368500

B -2.83310500 -1.63627100 -1.68714300

B -1.63627100 -2.83310500 -1.68714300

B 0.00000000 -3.21377900 -1.71368500

B 1.63627100 -2.83310500 -1.68714300

B 2.83310500 -1.63627100 -1.68714300

B 3.21377900 0.00000000 -1.71368500

B 2.83310500 1.63627100 -1.68714300

B 0.80829500 2.99766200 -3.16525700

B -0.80829500 2.99766200 -3.16525700

B -2.21077800 2.21077800 -3.16144600

B -2.99766200 0.80829500 -3.16525700

B -2.99766200 -0.80829500 -3.16525700

B -2.21077800 -2.21077800 -3.16144600

B -0.80829500 -2.99766200 -3.16525700

B 0.80829500 -2.99766200 -3.16525700

B 2.21077800 -2.21077800 -3.16144600

B 2.99766200 -0.80829500 -3.16525700

B 2.99766200 0.80829500 -3.16525700

B 2.21077800 2.21077800 -3.16144600

Ca 0.00000000 0.00000000 2.96607600

| *D*_4_*_h_* Ca©B_56_ (**B-2**) |
| --- |

B 0.80664100 3.00327800 2.97292600

B -0.80664100 3.00327800 2.97292600

B -2.20894000 2.20894000 2.97272200

B -3.00327800 0.80664100 2.97292600

B -3.00327800 -0.80664100 2.97292600

B -2.20894000 -2.20894000 2.97272200

B -0.80664100 -3.00327800 2.97292600

B 0.80664100 -3.00327800 2.97292600

B 2.20894000 -2.20894000 2.97272200

B 3.00327800 -0.80664100 2.97292600

B 3.00327800 0.80664100 2.97292600

B 2.20894000 2.20894000 2.97272200

B 1.62965500 2.82012500 1.49112400

B 0.00000000 3.23254000 1.51443800

B -1.62965500 2.82012500 1.49112400

B -2.82012500 1.62965500 1.49112400

B -3.23254000 0.00000000 1.51443800

B -2.82012500 -1.62965500 1.49112400

B -1.62965500 -2.82012500 1.49112400

B 0.00000000 -3.23254000 1.51443800

B 1.62965500 -2.82012500 1.49112400

B 2.82012500 -1.62965500 1.49112400

B 3.23254000 0.00000000 1.51443800

B 2.82012500 1.62965500 1.49112400

B 0.83705300 3.03144100 0.00000000

B -0.83705300 3.03144100 0.00000000

B -3.03144100 0.83705300 0.00000000

B -3.03144100 -0.83705300 0.00000000

B -0.83705300 -3.03144100 0.00000000

B 0.83705300 -3.03144100 0.00000000

B 3.03144100 -0.83705300 0.00000000

B 3.03144100 0.83705300 0.00000000

B 1.62965500 2.82012500 -1.49112400

B 0.00000000 3.23254000 -1.51443800

B -1.62965500 2.82012500 -1.49112400

B -2.82012500 1.62965500 -1.49112400

B -3.23254000 0.00000000 -1.51443800

B -2.82012500 -1.62965500 -1.49112400

B -1.62965500 -2.82012500 -1.49112400

B 0.00000000 -3.23254000 -1.51443800

B 1.62965500 -2.82012500 -1.49112400

B 2.82012500 -1.62965500 -1.49112400

B 3.23254000 0.00000000 -1.51443800

B 2.82012500 1.62965500 -1.49112400

B 0.80664100 3.00327800 -2.97292600

B -0.80664100 3.00327800 -2.97292600

B -2.20894000 2.20894000 -2.97272200

B -3.00327800 0.80664100 -2.97292600

B -3.00327800 -0.80664100 -2.97292600

B -2.20894000 -2.20894000 -2.97272200

B -0.80664100 -3.00327800 -2.97292600

B 0.80664100 -3.00327800 -2.97292600

B 2.20894000 -2.20894000 -2.97272200

B 3.00327800 -0.80664100 -2.97292600

B 3.00327800 0.80664100 -2.97292600

B 2.20894000 2.20894000 -2.97272200

Ca 0.00000000 0.00000000 0.00000000

| *C_s_* Ca©B_56_ (**B-4**) |
| --- |

B -0.96715000 -2.68273000 2.76744000

B -2.27062000 -1.76475000 2.70888000

B -3.45767000 -0.89162000 2.07890000

B -4.22187000 -0.32243000 0.79780000

B -4.22187000 -0.32243000 -0.79780000

B -3.45767000 -0.89162000 -2.07890000

B -2.27062000 -1.76475000 -2.70888000

B -0.96715000 -2.68273000 -2.76744000

B 0.25292000 -3.50711000 -2.13283000

B 0.97578000 -4.01306000 -0.79936000

B 0.97578000 -4.01306000 0.79936000

B 0.25292000 -3.50711000 2.13283000

B 0.59856000 -1.95892000 2.75119000

B -0.76685000 -1.00933000 2.96648000

B -2.10946000 -0.04488000 2.70147000

B -3.13480000 0.71195000 1.60708000

B -3.53412000 1.01548000 0.00000000

B -3.13480000 0.71195000 -1.60708000

B -2.10946000 -0.04488000 -2.70147000

B -0.76685000 -1.00933000 -2.96648000

B 0.59856000 -1.95892000 -2.75119000

B 1.64683000 -2.67919000 -1.62811000

B 2.01541000 -2.92390000 0.00000000

B 1.64683000 -2.67919000 1.62811000

B 0.77333000 -0.31534000 3.09967000

B -0.61360000 0.67143000 3.08799000

B -2.50426000 2.08700000 0.84388000

B -2.50426000 2.08700000 -0.84388000

B -0.61360000 0.67143000 -3.08799000

B 0.77333000 -0.31534000 -3.09967000

B 2.74241000 -1.65363000 -0.85096000

B 2.74241000 -1.65363000 0.85096000

B 2.27747000 0.44834000 2.80505000

B 0.95220000 1.40559000 3.20492000

B -0.38712000 2.33961000 2.79973000

B -1.37233000 3.08464000 1.64251000

B -1.62952000 3.30321000 0.00000000

B -1.37233000 3.08464000 -1.64251000

B -0.38712000 2.33961000 -2.79973000

B 0.95220000 1.40559000 -3.20492000

B 2.27747000 0.44834000 -2.80505000

B 3.28681000 -0.23562000 -1.63497000

B 3.61632000 -0.41898000 0.00000000

B 3.28681000 -0.23562000 1.63497000

B 2.45556000 2.12669000 3.00984000

B 1.14028000 3.06176000 3.00919000

B 0.01136000 3.88584000 2.21277000

B -0.60544000 4.36129000 0.80715000

B -0.60544000 4.36129000 -0.80715000

B 0.01136000 3.88584000 -2.21277000

B 1.14028000 3.06176000 -3.00919000

B 2.45556000 2.12669000 -3.00984000

B 3.60379000 1.33133000 -2.21692000

B 4.25523000 0.89641000 -0.80902000

B 4.25523000 0.89641000 0.80902000

B 3.60379000 1.33133000 2.21692000

Ca -1.39770000 -2.91060000 0.00000000

| *D*_4_*_h_* B_56_^2−^ |
| --- |

B 0.80590300 3.00529100 2.96219300

B -0.80590300 3.00529100 2.96219300

B -2.20152100 2.20152100 2.95950900

B -3.00529100 0.80590300 2.96219300

B -3.00529100 -0.80590300 2.96219300

B -2.20152100 -2.20152100 2.95950900

B -0.80590300 -3.00529100 2.96219300

B 0.80590300 -3.00529100 2.96219300

B 2.20152100 -2.20152100 2.95950900

B 3.00529100 -0.80590300 2.96219300

B 3.00529100 0.80590300 2.96219300

B 2.20152100 2.20152100 2.95950900

B 1.63698500 2.83032500 1.47965300

B 0.00000000 3.20086500 1.49019800

B -1.63698500 2.83032500 1.47965300

B -2.83032500 1.63698500 1.47965300

B -3.20086500 0.00000000 1.49019800

B -2.83032500 -1.63698500 1.47965300

B -1.63698500 -2.83032500 1.47965300

B 0.00000000 -3.20086500 1.49019800

B 1.63698500 -2.83032500 1.47965300

B 2.83032500 -1.63698500 1.47965300

B 3.20086500 0.00000000 1.49019800

B 2.83032500 1.63698500 1.47965300

B 0.84590600 3.12611500 0.00000000

B -0.84590600 3.12611500 0.00000000

B -3.12611500 0.84590600 0.00000000

B -3.12611500 -0.84590600 0.00000000

B -0.84590600 -3.12611500 0.00000000

B 0.84590600 -3.12611500 0.00000000

B 3.12611500 -0.84590600 0.00000000

B 3.12611500 0.84590600 0.00000000

B 1.63698500 2.83032500 -1.47965300

B 0.00000000 3.20086500 -1.49019800

B -1.63698500 2.83032500 -1.47965300

B -2.83032500 1.63698500 -1.47965300

B -3.20086500 0.00000000 -1.49019800

B -2.83032500 -1.63698500 -1.47965300

B -1.63698500 -2.83032500 -1.47965300

B 0.00000000 -3.20086500 -1.49019800

B 1.63698500 -2.83032500 -1.47965300

B 2.83032500 -1.63698500 -1.47965300

B 3.20086500 0.00000000 -1.49019800

B 2.83032500 1.63698500 -1.47965300

B 0.80590300 3.00529100 -2.96219300

B -0.80590300 3.00529100 -2.96219300

B -2.20152100 2.20152100 -2.95950900

B -3.00529100 0.80590300 -2.96219300

B -3.00529100 -0.80590300 -2.96219300

B -2.20152100 -2.20152100 -2.95950900

B -0.80590300 -3.00529100 -2.96219300

B 0.80590300 -3.00529100 -2.96219300

B 2.20152100 -2.20152100 -2.95950900

B 3.00529100 -0.80590300 -2.96219300

B 3.00529100 0.80590300 -2.96219300

B 2.20152100 2.20152100 -2.95950900
